# Achados Ecocardiográficos em Crianças de Pacientes com Diagnóstico de Síndrome do *PRKAG2*

**DOI:** 10.36660/abc.20230531

**Published:** 2024-08-15

**Authors:** Dinamar Amador dos Santos, Igor de Souza, Alice Pinheiro Barbosa, Eduardo Back Sternick, José Luiz Barros Pena

**Affiliations:** 1 Faculdade Ciências Médicas de Minas Gerais Belo Horizonte MG Brasil Faculdade Ciências Médicas de Minas Gerais, Belo Horizonte, MG – Brasil; 2 Hospital Felício Rocho - Ecocardiografia Belo Horizonte MG Brasil Hospital Felício Rocho - Ecocardiografia, Belo Horizonte, MG – Brasil

**Keywords:** Síndrome *PRKAG2*/genética, Criança, Doença de depósito de glicogênio/complicações, Ecocardiografia/métodos

## Abstract

**Fundamento:**

A síndrome do *PRKAG2* tipicamente se manifesta na adolescência e início da idade adulta, cursando com hipertrofia ventricular esquerda, arritmias e risco de morte súbita. O achado de marcadores ecocardiográficos antes da manifestação clínica nos filhos de pais acometidos pela doença pode facilitar a estratégia de prevenção e planejamento terapêutico para esse grupo de pacientes.

**Objetivo:**

Identificar a existência de achados ecocardiográficos que se manifestem precocemente nos filhos de pais acometidos por síndrome do *PRKAG2*, enquanto ainda assintomáticos.

**Métodos:**

Estudo observacional transversal em que sete participantes, filhos de pais com diagnóstico estabelecido de síndrome do *PRKAG2*, com idades entre 9 meses e 12 anos e diagnóstico genético comprovado, foram submetidos à ecocardiografia convencional e por técnicas avançadas, tendo seus achados comparados aos de grupo controle composto por sete voluntários pareados por sexo e idade, hígidos do ponto de vista cardiovascular. Um valor de p < 0,05 foi considerado significante.

**Resultados:**

A ecocardiografia convencional mostrou valores aumentados com significância estatística no grupo caso para átrio esquerdo, septo interventricular, parede posterior do ventrículo esquerdo, massa ventricular indexada e espessura relativa da parede (p < 0,05). O *strain* sistólico longitudinal global obtido pelo ecocardiograma bidimensional não mostrou diferença estatisticamente significativa entre os grupos caso e controle. Nenhum dos parâmetros ao ecocardiograma tridimensional apresentou significância estatística entre os grupos.

**Conclusão:**

Crianças diagnosticadas com *PRKAG2* demonstraram achados ecocardiográficos indicativos de tendência à hipertrofia cardíaca. A ecocardiografia pode ser uma ferramenta útil na avaliação e seguimento desse grupo de pacientes, antes do início de manifestações clínicas.

**Figure:**
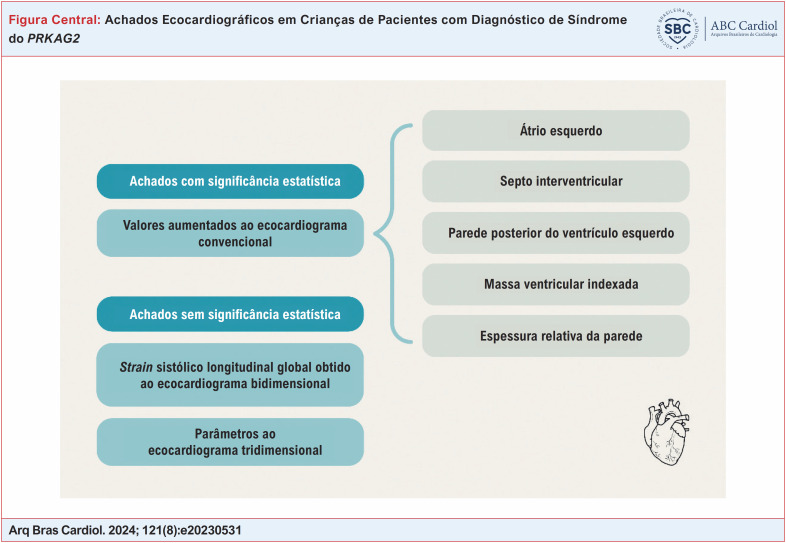


## Introdução

A inusitada associação entre cardiomiopatia hipertrófica e pré-excitação ventricular em indivíduos ao final da adolescência e início da idade adulta aventou a hipótese da ocorrência de alguma outra anormalidade de cunho genético. A confirmação de que alterações no lócus 7q3 se associavam a mutações no gene *PRKAG2* elucidaram o substrato da decorrente síndrome autossômica dominante de caráter familiar.^1,2^

A subunidade γ2 do gene *PRKAG2* desempenha papel regulatório na síntese da proteína quinase ativada por monofosfato de adenosina (AMPK). A disfunção dessa proteína resulta em acúmulo de glicogênio nos miócitos cardíacos, levando à hipertrofia ventricular esquerda.^3^

A despeito da manifestação fenotípica similar, a síndrome do *PRKAG2* difere da cardiomiopatia hipertrófica em vários níveis.^4^ Sendo doença de depósito do glicogênio, seu padrão histológico não guarda relação com o desarranjo miofibrilar observado nas cardiomiopatias sarcoméricas.^5^ Podemos observar aumento do diâmetro dos cardiomiócitos e pronunciada vacualização. A microscopia eletrônica demonstra grande quantidade de depósito de grânulos de glicogênio no citoplasma, principalmente na região perinuclear. Rara, ela tende à piora progressiva com a idade, com deterioração do sistema de condução motivando implante de marca-passo tipicamente entre a terceira e a quinta décadas de vida.^6^

A prevalência desta síndrome não está totalmente determinada, pois muitos casos não são adequadamente diagnosticados, sendo rotulados como cardiomiopatia hipertrófica familial. Estima-se que 2% a 5% dos casos de cardiopatia hipertrófica são decorrentes da mutação do gene *PRKAG2*.

Embora a ocorrência de sintomas tipicamente se inicie na segunda e terceira décadas, foram descritos casos de início já no período neonatal, com rápida e catastrófica deterioração da função cardíaca.^7^ Seu diverso espectro de apresentação, que pode incluir síncope, dor torácica, insuficiência cardíaca, mialgia e epilepsia, dificulta o diagnóstico diferencial com outras doenças de depósito com repercussão cardíaca. Morte súbita cardíaca pode ser a primeira manifestação.

O fato de não possuir abordagem terapêutica específica, as dificuldades no diagnóstico diferencial e a importante morbimortalidade ocasionada em pacientes jovens motivam a busca por estratégias de detecção precoce da síndrome do *PRKAG2*. Estudos recentes consolidaram a importância da ecocardiografia como estratégia válida, de caráter não invasivo e amplamente disponível. Sob tal prisma, o uso de técnicas avançadas, como índices de deformação miocárdica (*strain/strain rate*) pela técnica do *speckle tracking* (STE) e ecocardiografia tridimensional (3D), mostraram-se úteis na avaliação da estrutura e função cardíaca nesse grupo de pacientes.^8^

A busca por marcadores ecocardiográficos que permitem elaborar estratégias que se antecipem às manifestações clínicas da síndrome do *PRKAG2*, somada à escassez de estudos que correlacionem parâmetros ecocardiográficos em pacientes na infância, motivou a realização do presente estudo.

## Métodos

### Participantes e protocolo do estudo

Estudo clínico observacional transversal, comparativo entre crianças comprovadamente portadoras da síndrome do *PRKAG2*, filhos de pais com a doença geneticamente comprovada em acompanhamento ambulatorial em nossa instituição e pacientes hígidos do ponto de vista cardiovascular, realizado entre janeiro de 2018 e março de 2023. Nossa amostra foi de conveniência, devido à raridade da mutação estudada. Sete participantes, de sete famílias diferentes, com idades de 9 meses a 12 anos, tiveram suas medidas ecocardiográficas comparadas às de sete participantes pareados por sexo e idade, hígidos do ponto de vista cardiovascular. As crianças do grupo controle foram consideradas normais em exame de rotina realizado por pediatra. Todas as crianças e pais apresentaram a mutação Arg302Gln através do mapeamento genético sequencial pelo método de Sanger. Todos os participantes do estudo foram submetidos a exame clínico, eletrocardiográfico com 12 derivações e ecocardiograma transtorácico. Nosso estudo foi realizado seguindo as diretrizes das Boas Práticas Clínicas e aprovado pelo Comitê de Ética em Pesquisa da instituição, sob o número 17616119.0.0000.5134. O termo de consentimento livre e esclarecido foi obtido dos responsáveis pelos participantes, e termo de assentimento foi obtido dos participantes com idade a partir de 6 anos de idade.

### Análise ecocardiográfica

Todos os pacientes foram submetidos a exame ecocardiográfico transtorácico completo, seguindo as recomendações da Sociedade Americana de Ecocardiografia (ASE) e da Associação Europeia de Imagem Cardiovascular (EACVI).^9^ Todos os estudos foram realizados utilizando um sistema ecocardiográfico disponível comercialmente, equipamento Vivid E9 (GE Healthcare, Horten, Noruega). O exame incluiu o modo-M, medidas através do ecocardiograma bidimensional (2D), STE 2D e medidas 3D. Todos os exames foram feitos por um único ecocardiografista experiente, com título de especialista pelo Departamento de Imagem Cardiovascular da Sociedade Brasileira de Cardiologia.

A massa miocárdica foi medida através do modo-M direcionado pelo 2D, utilizando o método de convenção de Penn e a fórmula da ASE.^10^ A espessura relativa da parede (ERP) foi calculada como 2 vezes a razão entre a espessura da parede posterior do ventrículo esquerdo (VE) dividida pelo diâmetro diastólico do VE ao final da diástole. Foi considerada hipertrofia concêntrica se essa medida fosse igual ou superior a 0,42. Foi medida a relação do volume diastólico final dividido pela massa total do VE em todas as crianças.

Através do fluxo transmitral ao Doppler pulsátil foram medidas as velocidades de pico do enchimento precoce (onda E) e tardio (onda A), a relação E/A das velocidades de pico e tempo de desaceleração da onda E. As velocidades do anel valvar mitral septal e lateral (onda e’) foram obtidas através do Doppler tecidual, em corte apical quatro câmaras.

Janelas apicais de 2, 3 e 4 câmaras, foram utilizadas para obtenção do 2D *strain* sistólico longitudinal do VE. A geração de imagem paramétrica do *strain* miocárdico foi possível pela integração de método automatizado. O s*train* longitudinal de cada segmento foi medido e expresso em *bull's eye*. O *software* calculou o *strain* longitudinal global como a média dos *strains* regionais ao longo de todo VE. Os dados ecocardiográficos 3D foram obtidos através de 6 batimentos consecutivos, eletrocardiograficamente monitorados, para cálculo do volume total.

O traçado automático das bordas endocárdicas e epicárdicas permitiu a obtenção de fração de ejeção ao fim da sístole e ao fim da diástole, débito cardíaco, índice de esfericidade, massa do VE e parâmetros 3D de deformação miocárdica: *strain* longitudinal global, *strain* global circunferencial, *area strain* global e *strain* global radial. Os dados obtidos foram exportados para uma estação de trabalho EchoPAC versão 112.1.3, GE Healthcare para análise *offline*.

### Análise estatística

Os dados foram agrupados em tabelas de frequência, com as frequências absolutas e suas respectivas porcentagens, assim como as medidas descritivas (mediana, intervalo interquartil [percentis 25% e 75%] para os dados quantitativos). A avaliação da normalidade foi realizada através do teste de Shapiro-Wilk. Para a comparação dos dados categóricos foi utilizado o teste de Fisher, e para a comparação de variáveis quantitativas foi utilizado o teste de Mann-Whitney, por não apresentarem distribuição normal. Em todos os testes, foram consideradas significativas comparações cujo valor-p foi inferior a 5%. O *software* utilizado para as análises foi o SPSS versão 25.0.

## Resultados

Nenhuma criança apresentou sintomas ou queixas cardiológicas. Em um paciente foi detectado sopro sistólico em foco aórtico de grau I, com bulhas normofonéticas. O eletrocardiograma basal demonstrou ritmo sinusal em todos os pacientes. Nenhum deles apresentou síndrome de pré-excitação. Uma das crianças do grupo caso apresentou intervalo Pr curto e outra bloqueio de ramo direito grau II no eletrocardiograma basal.

A Tabela 1 apresenta as características demográficas e clínicas dos grupos caso e controle estudados. Os grupos são homogêneos quanto ao sexo, idade, peso, altura, superfície corpórea e frequência cardíaca medida durante o exame 3D.

**Tabela 1:** Características demográficas e clínicas de acordo com os grupos estudados

Variáveis		Grupo		Valor p
		Caso (n = 7)	Controle (n = 7)	
**Sexo**				
	Feminino	4 (57,1%)	4 (57,1%)	>0,999^f^
	Masculino	3 (42,9%)	3 (42,9%)	
**Idade**				
	Mediana (IQR)	6,0 (2,0 - 8,0)	6,0 (2,0 - 8,0)	>0,999^mw^
**Peso**				
	Mediana (IQR)	22,0 (13,0 - 39,0)	18,0 (13,3 - 27,0)	0,710^mw^
**Altura**				
	Mediana (IQR)	1,20 (1,00 - 1,42)	1,10 (1,05 - 1,25)	0,805^mw^
**Superfície corpórea**				
	Mediana (IQR)	0,86 (0,60 - 1,24)	0,74 (0,70 - 0,97)	0,805^mw^
**FC 3D**				
	Mediana (IQR)	56 (52 - 88)	80 (68 - 98)	0,165^mw^

f: teste exato de Fisher; FC 3D: frequência cardíaca durante ecocardiograma tridimensional; IQR: interquartil; mw: teste de Mann-Whitney.

**Figura 1:**
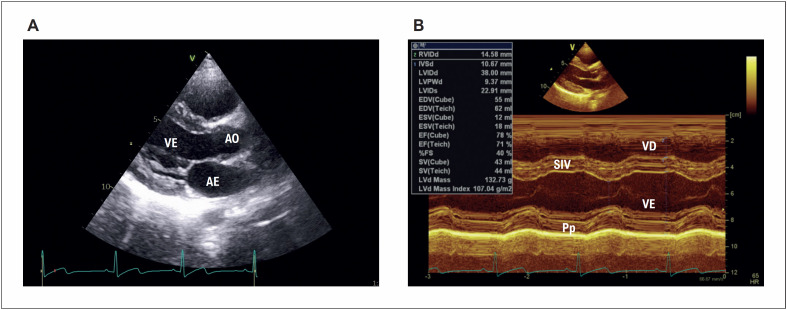
Em A) corte paraesternal eixo longitudinal do ventrículo esquerdo. Em B) modo-M orientado pela imagem bidimensional, onde foram feitas as medidas das câmaras cardíacas, massa miocárdica e função sistólica do VE. AE: átrio esquerdo; AO: aorta; VE: ventrículo esquerdo; Pp: parede posterior em diástole; SIV: septo interventricular em diástole; VD: ventrículo direito.

Na avaliação dos parâmetros ecocardiográficos convencionais foi observada diferença significativa para diâmetro anteroposterior do átrio esquerdo, espessura diastólica do septo interventricular e da parede posterior do VE, massa miocárdica do VE indexada pela superfície corpórea, relação volume diastólico final dividido pela massa total (VDF/M) e espessura relativa da parede do VE (ERP) (Figura 1). Para todas essas medidas, a mediana foi maior nos casos, exceto para VDF/M que mostrou maior mediana para o grupo controle (Tabela 2).

**Tabela 2:** Avaliação dos parâmetros ecocardiográficos convencionais de acordo com os grupos estudados

Variáveis		Grupo		Valor p
		Casos (n = 7)	Controles (n = 7)	
**Ao**				
	Mediana (IQR)	23 (17 - 23)	18 (17 - 22)	0,259
**AE**				
	Mediana (IQR)	27 (19 - 30)	18 (16 - 23)	0,026
**VD**				
	Mediana (IQR)	14 (12 - 16)	12 (10 - 16)	0,383
**VE sist**				
	Mediana (IQR)	23 (19 - 24)	23 (20 - 26)	0,383
**VE diast**				
	Mediana (IQR)	38 (31 - 42)	36 (30 - 42)	0,535
**SIV**				
	Mediana (IQR)	8 (6 - 10)	6 (4 - 6)	0,017
**Ppost**				
	Mediana (IQR)	8 (6 - 9)	5 (4 - 5)	0,007
**Índice**				
	Mediana (IQR)	1 (1 - 1,2)	1 (1 - 1,2)	0,902
**VDF**				
	Mediana (IQR)	61 (37 - 78)	45 (36 - 61)	0,209
**VSF**				
	Mediana (IQR)	18 (11 - 21)	14 (11 - 21)	0,620
**VS**				
	Mediana (IQR)	27 (21 - 44)	30 (22 - 43)	>0,999
**FE**				
	Medianª (IQR)	72 (71 - 76)	67 (62 - 76)	0,165
**FS %**				
	Mediana (IQR)	41 (39 - 45)	38 (33 - 42)	0,138
**Massa miocárdica VE**				
	Mediana (IQR)	103 (27,6 - 133)	30 (20,6 - 64)	0,128
**Massa indexada VE**				
	Mediana (IQR)	96 (67,3 - 107)	43 (37,8 - 54)	0,007
**VDF/M**				
	Mediana (IQR)	0,59 (0,57 - 0,8)	1,1 (1,04 - 1,75)	0,004
**ERP**				
	Mediana (IQR)	0,42 (0,32 - 0,44)	0,28 (0,24 - 0,33)	0,007
**Onda E valva mitral**				
	Mediana (IQR)	1,08 (0,86 - 1,33)	1 (0,89 - 1,2)	0,620
**Onda A valva mitral**				
	Mediana (IQR)	0,36 (0,31 - 0,49)	0,38 (0,34 - 0,42)	0,710
**Relação E/A mitral**				
	Mediana (IQR)	2,7 (2,2 - 3,4)	2,48 (2,3 - 2,6)	0,902
**Tempo desaceleração E**				
	Mediana (IQR)	160 (151 - 174)	174 (152 - 180)	0,383
**Onda e' septal**				
	Mediana (IQR)	12 (11 - 13)	12 (10 - 14)	0,805
**Relação onda E/onda e'**				
	Mediana (IQR)	8,1 (7,2 - 10)	8,3 (5,2 - 8,6)	0,535
**IT pico velocidade**				
	Mediana (IQR)	2,28 (2,08 - 2,41)	2,3 (1,94 - 2,35)	0,556
**PSAP**				
	Mediana (IQR)	26 (23 - 29)	26 (20 - 27)	0,413

AE: átrio esquerdo; Ao: aorta; ERP: espessura relativa da parede; FE: fração de ejeção; FS: porcentagem de encurtamento do VE; IT: insuficiência tricúspide; PSAP: pressão sistólica da artéria pulmonar; Ppost: espessura da parede posterior do ventrículo esquerdo em diástole; SIV: espessura do septo interventricular em diástole; VD: ventrículo direito; VDF: volume diastólico final do ventrículo esquerdo; VE sist: diâmetro do ventrículo esquerdo em sístole; VE diast: diâmetro do ventrículo esquerdo em diástole; VS: volume sistólico ejetivo; VSF: volume sistólico final.

* Teste de Mann-Whitney.

Quanto aos parâmetros ecocardiográficos ao exame 3D não foi observada diferença estatisticamente significativa (Figura Central). Os dados estão enumerados na Tabela 3.

**Tabela 3:** Avaliação dos parâmetros ecocardiográficos tridimensionais de acordo com os grupos estudados

Variáveis		Grupo		Valor p
		Casos (n = 7)	Controles (n = 7)	
**Volume diastólico final 3D**				
	Mediana (IQR)	45 (24 - 57)	42 (26 - 59)	>0,999
**Volume sistólico final 3D**				
	Mediana (IQR)	17 (10 - 22)	16 (11 - 24)	>0,999
**Volume sistólico 3D**				
	Mediana (IQR)	27 (15 - 35)	26 (16 - 35)	>0,999
**Fração ejeção 3D**				
	Mediana (IQR)	62 (60 - 63)	62 (59 - 63)	>0,999
**Débito cardíaco 3D**				
	Mediana (IQR)	1,5 (1,3 - 2)	1,6 (1,5 - 2,1)	0,456
**Massa miocárdica g 3D**				
	Mediana (IQR)	80 (30 - 96)	54 (31 - 60)	0,165
**Massa miocárdica g/m^2^ 3D**				
	Mediana (IQR)	87,7 (59 - 112)	61 (44 - 68)	0,165
**Índice esfericidade 3D**				
	Mediana (IQR)	0,51 (0,45 - 0,59)	0,6 (0,56 - 0,6)	0,165

3D: ecocardiograma tridimensional; IQR: interquartil.

**Figura 2:**
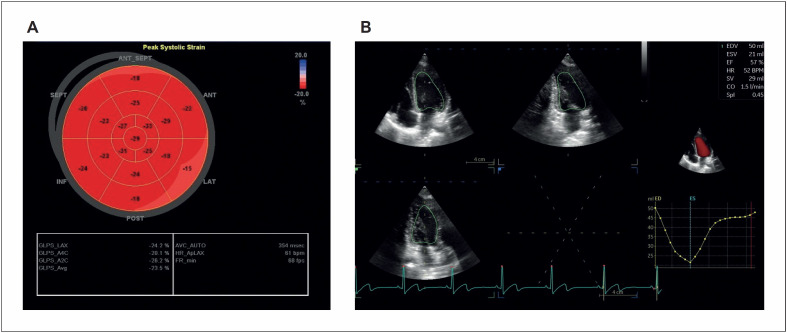
Em A) representação paramétrica em bull's eye do strain sistólico longitudinal obtido ao exame bidimensional. Em B) exame tridimensional, onde foram obtidos os volumes diastólico e sistólico finais, fração de ejeção, débito cardíaco e índice de esfericidade.

Em relação aos índices de deformação miocárdica obtidos pela técnica de STE, tanto na modalidade 2D quanto na 3D, também não foi detectada alteração estatisticamente significativa (Tabela 4, Figura 2).

**Tabela 4:** Índices de deformação miocárdica através do speckle tracking bi e tridimensional

Variáveis		Grupo		Valor p
		Casos (n = 7)	Controles (n = 7)	
SGL 3D				
	Mediana (IQR)	18 (17 - 21)	19 (18 - 21)	0,259
SGR 3D				
	Mediana (IQR)	51 (47 - 56)	52 (48 - 56)	0,620
SGC 3D				
	Mediana (IQR)	17 (15 - 28)	20 (18,8 - 21)	0,128
Area strain 3D				
	Mediana (IQR)	31 (29 - 34)	32 (30 - 36)	0,259
Plano 3 câmaras 2D				
	Mediana (IQR)	18,3 (18,1 - 20,4)	19,2 (19,1 - 22,4)	0,097
Plano 4 câmaras 2D				
	Mediana (IQR)	19,9 (19,1 - 20,1)	20,1 (19,6 - 20,3)	0,318
Plano 2 câmaras 2D				
	Mediana (IQR)	21,4 (20,8 - 24,9)	22,8 (21,2 - 23,8)	0,710
SGL 2D				
	Mediana (IQR)	19,7 (19,3 - 21)	20,7 (20,2 - 22,6)	0,318

IQR: interquartil; SGC 3D: *strain* global circunferencial ao ecocardiograma tridimensional; SGL 2D: *strain* global longitudinal ao ecocardiograma bidimensional; SGL 3D: *strain* global longitudinal ao ecocardiograma tridimensional; SGR 3D: *strain* global radial ao ecocardiograma tridimensional.

## Discussão

Desde a descrição por Gollob et al., em 2001,^1^ do gene PRKAG2 como o causador da associação sindrômica entre hipertrofia ventricular esquerda, pré-excitação, progressiva deterioração do sistema de condução e morte súbita, os relatos acumulados nas últimas duas décadas permitiram reconhecer o amplo escopo de manifestações clínicas que podem se associar à síndrome do *PRKAG2*, tão diversas como mialgias, convulsões, síncope, bloqueio atrioventricular progressivo e insuficiência cardíaca.^6,7,11^

Não obstante, a evolução para falência cardíaca franca em primeiros anos de vida representa exceção.^7^ Na maioria dos casos descritos, observa-se evolução assintomática ou com sintomatologia discreta e inespecífica até o final da adolescência e início da idade adulta.^6^

A síndrome do *PRKAG2* decorre da mutação autossômica dominante, de caráter familiar. Leva à desregulação da AMPK, com consequente desregulação metabólica que leva ao progressivo acúmulo de glicogênio. Sua ocorrência é rara, e seu correto diagnóstico requer estudo genético e extensa avaliação cardiológica.^11,12^

O aspecto eletrocardiográfico nessa doença apresenta intervalo Pr curto na maioria dos casos, bloqueio de ramo direito, bloqueios atrioventriculares ou sinoatriais. Alta voltagem nos complexos QRS com anormalidades de repolarização ventricular pode ser observada mesmo na ausência de hipertrofia do VE ao ecocardiograma.^13^

A caracterização das alterações ecocardiográficas expressas no curso da doença no adulto tem sido pesquisada.^12,13^ Para além do uso da ecocardiografia convencional, o auxílio da modalidade 3D e da avaliação da deformação miocárdica permitiram melhor interpretação das alterações progressivas de morfologia e função cardíaca que se associam ao curso da doença.^8,14,15^ No entanto, o foco da maioria dos estudos que se utilizam da ecocardiografia tem sido em pacientes adultos, com manifestações da doença já evidentes.

Tendo em perspectiva o caráter autossômico dominante da mutação do *PRKAG2*, o caráter cumulativo e progressivo decorrente da disfunção da AMPK e o curso natural da doença, é razoável supor que as alterações ecocardiográficas decorrentes do acúmulo de glicogênio se iniciem já na infância, gerando indicadores cada vez mais evidentes do curso evolutivo dessa entidade.

O presente estudo procura comparar os estudos ecocardiográficos de filhos de pacientes com diagnóstico com *PRKAG2*, com idade de 9 meses a 12 anos, pareados com indivíduos hígidos de grupo controle. Tomou-se o cuidado de que os participantes de pesquisa fossem pareados com indivíduos do mesmo sexo. Tendo em vista a relativa escassez de padronização das referências nas medidas ecocardiográficas em crianças, especialmente quando se considera as medidas obtidas em técnicas avançadas, tal pareamento teve por objetivo minorar as diferenças que não decorressem de manifestações cardíacas de mutação do *PRKAG2*. O tamanho exíguo da amostra se deve à raridade da doença.

À ecocardiografia convencional, as medidas do átrio esquerdo, septo interventricular, parede posterior do VE, massa indexada e ERP foram maiores do que as encontradas no grupo controle, mostrando significância estatística (p < 0,05).

Os achados acima descritos corroboram a esperada tendência de que nesta doença – caracterizada pelo acúmulo progressivo de glicogênio nos cardiomiócitos – alterações relacionadas à progressão para um padrão de hipertrofia ventricular já se encontrem muitos anos antes da manifestação clínica da doença.^16^ Ademais, o papel do ecocardiograma – ferramenta não invasiva e de ampla disponibilidade – no acompanhamento desses pacientes pode se mostrar viável no intuito de antecipar-se às manifestações clínicas nos filhos de acometidos por *PRKAG2*.

Embora a ecocardiografia 3D e a avaliação dos índices de deformação cardíaca já se tenham demonstrado como relevantes instrumentos de complementação à análise por ecocardiograma convencional, em nosso estudo não detectamos alterações significativas em relação ao grupo controle. Uma explicação possível seria de que o modo-M orientado pelo exame 2D apresenta uma maior resolução temporal, além de ser uma modalidade efetiva em registrar múltiplos ciclos cardíacos.^17^

O tamanho pequeno da amostra, o caráter transversal do estudo, a raridade da doença e a heterogeneidade dos fenótipos relacionados à síndrome do *PRKAG2*, assim como poucos estudos de valores de referência para crianças das novas tecnologias ecocardiográficas, constituem limitações do estudo.

## Conclusão

Crianças portadoras de variantes patogênicas em *PRKAG2* e filhos de pais com essa mesma doença já apresentam tendência ao aumento da espessura das paredes do VE em relação ao grupo controle. O acompanhamento ecocardiográfico, desde a infância, dos filhos de pais acometidos pela síndrome do *PRKAG2* pode ser útil na antecipação de manifestações da doença, permitindo melhor planejamento do acompanhamento terapêutico e prognóstico. São necessários maiores estudos, de caráter longitudinal e focados em crianças, para a melhor compreensão da evolução da doença e identificação de achados ecocardiográficos úteis para o acompanhamento desse grupo especial de pacientes.
