# Supplementary material for: Achados Ecocardiográficos em Crianças de Pacientes com Diagnóstico de Síndrome do PRKAG2
Source: Arq Bras Cardiol. 2024 Aug 15;121(8):e20230531. [Article in Portuguese] doi: 10.36660/abc.20230531 (PMC11495819; doi:10.36660/abc.20230531)
Supplement: Translation

# Translation: Echocardiographic Findings in Children of Patients Diagnosed with *PRKAG2* Syndrome

**DOI:** 10.36660/abc.20230531i

**Authors:** Dinamar Amador dos Santos, Igor de Souza, Alice Pinheiro Barbosa, Eduardo Back Sternick, José Luiz Barros Pena

**Affiliations:** 1 Belo Horizonte MG Brazil Faculdade Ciências Médicas de Minas Gerais, Belo Horizonte, MG – Brazil; 2 Belo Horizonte MG Brazil Hospital Felício Rocho - Ecocardiografia, Belo Horizonte, MG – Brazil

**Keywords:** *PRKAG2* Syndrome/genetics, Child, Glycogen Storage Disease/complications, Echocardiography/methods

## Abstract

**Background:**

*PRKAG2* syndrome typically manifests in adolescence and early adulthood, progressing with left ventricular hypertrophy, arrhythmias, and risk of sudden death. Findings of echocardiographic markers before clinical manifestation in children of patients affected by the disease can facilitate prevention strategies and therapeutic planning for this patient group.

**Objective:**

To identify the existence of echocardiographic findings that manifest early in children of parents affected by *PRKAG2* syndrome, while they are still asymptomatic.

**Methods:**

In this cross-sectional observational study, 7 participants who were children of parents with established diagnosis of *PRKAG2* syndrome, between the ages of 9 months and 12 years, with proven genetic diagnosis, underwent conventional and advanced echocardiography. Their findings were compared to those of a control group composed of 7 age- and sex-matched volunteers who were healthy from a cardiovascular point of view. P values < 0.05 were considered significant.

**Results:**

Conventional echocardiography showed statistically significantly higher values in the case group for left atrium, interventricular septum, left ventricular posterior wall, indexed ventricular mass, and relative wall thickness (p < 0.05). Global longitudinal systolic strain on 2-dimensional echocardiography did not show statistical significance between the case and control groups. None of the parameters on 3-dimensional echocardiography showed statistical significance between groups.

**Conclusion:**

Children diagnosed with *PRKAG2* showed echocardiographic findings indicative of a tendency toward cardiac hypertrophy. Echocardiography can be a useful tool in the evaluation and follow-up of this patient group before the onset of clinical manifestations.

## Introduction

The rare association between hypertrophic cardiomyopathy and ventricular pre-excitation in individuals at the end of adolescence and early adulthood raised the hypothesis that there was another genetic abnormality. The confirmation that changes in the 7q3 locus were associated with mutations in the *PRKAG2* gene clarified the substrate of the resulting autosomal dominant familial syndrome.^1,2^

The γ2 subunit of the *PRKAG2* gene plays a regulatory role in the synthesis of adenosine monophosphate-activated protein kinase (AMPK). Dysfunction of this protein results in glycogen accumulation in cardiac myocytes, leading to left ventricular (LV) hypertrophy.^3^

Although it has similar phenotypic manifestation, *PRKAG2* syndrome differs from hypertrophic cardiomyopathy on multiple levels.^4^ As it is a glycogen storage disease, its histological pattern is not related to the myofibrillar disarray observed in sarcomeric cardiomyopathies.^5^ We can observe an increase in cardiomyocyte diameter and pronounced vacuolization. Electron microscopy demonstrates a large quantity of glycogen granules deposited in the cytoplasm, mainly in the perinuclear region. The disease is rare, and it tends to progressively worsen with age, with deterioration of the conduction system leading to pacemaker implantation, typically between the third and fifth decades of life.^6^

The prevalence of this syndrome has not been fully determined, as many cases are not adequately diagnosed and are labeled as familial hypertrophic cardiomyopathy. It is estimated that 2% to 5% of cases of hypertrophic heart disease are due to a mutation in the *PRKAG2* gene.

Although the occurrence of symptoms typically begins in the second and third decades of life, cases of onset in the neonatal period have been described, with rapid and catastrophic deterioration of cardiac function.^7^ Its diverse spectrum of presentation, which may include syncope, chest pain, heart failure, myalgia, and epilepsy, complicates differential diagnosis with other storage diseases with cardiac repercussions. Sudden cardiac death may be the first manifestation.

Due to the fact that there is no specific therapeutic approach, the difficult differential diagnosis, and the important morbidity and mortality in young patients have led to the search for strategies for early detection of *PRKAG2* syndrome. Recent studies have consolidated the importance of echocardiography as a valid, non-invasive, and widely available strategy. From this viewpoint, the use of advanced techniques, such as indices of myocardial deformation (strain/strain rate) on speckle tracking and three-dimensional (3D) echocardiography, have shown to be useful in evaluating cardiac structure and function in this patient group.^8^

The search for echocardiographic markers that make it possible to develop strategies that anticipate clinical manifestations of *PRKAG2* syndrome, combined with the scarcity of studies that correlate patients’ echocardiographic parameters during childhood, motivated us to carry out the present study.

## Methods

### Study participants and protocol

This cross-sectional observational clinical study compared children with proven diagnosis of *PRKAG2* syndrome, whose parents had genetically proven disease, undergoing outpatient follow-up at our institution, and patients who were healthy from a cardiovascular point of view, between January 2018 and March 2023. We used a convenience sample, due to the rarity of the studied mutation. We compared echocardiographic measurements of 7 participants, from 7 different families, aged 9 months to 12 years, to those of 7 sex- and age-matched participants, who were healthy from a cardiovascular point of view. The children in the control group were considered normal in a routine examination carried out by a pediatrician. All children and their parents presented the Arg302Gln mutation on Sanger gene sequencing. All study participants underwent clinical examination, 12-lead electrocardiography, and transthoracic echocardiography. Our study was carried out following Good Clinical Practices guidelines and approved by the institution's Research Ethics Committee, under number 17616119.0.0000.5134. The free and informed consent form was signed by the participants’ guardians, and assent was obtained from participants aged 6 years and over.

### Echocardiography analysis

All patients underwent complete transthoracic echocardiogram, following the recommendations of the American Society of Echocardiography (ASE) and the European Association of Cardiovascular Imaging (EACVI).^9^ All exams were performed using a commercially available echocardiographic system, Vivid E9 equipment (GE Healthcare, Horten, Norway). The examination included M-mode, two-dimensional (2D) echocardiographic measurements, 2D speckle tracking, and 3D measurements. All exams were performed by a single experienced echocardiographer, recognized as a specialist by the Department of Cardiovascular Imaging of the Brazilian Society of Cardiology.

Myocardial mass was measured through 2D-guided M-mode, using the Penn convention method and the ASE formula.^10^ Relative wall thickness (RWT) was calculated as 2 times the ratio of LV posterior wall thickness divided by LV diastolic diameter at end-diastole. Concentric hypertrophy was considered when the measurement was greater than or equal to 0.42. The ratio of end-diastolic volume divided by total LV mass was measured in all children.

Through transmitral flow on pulsed Doppler, the peak velocities of early (E wave) and late (A wave) filling, the ratio of peak velocities E/A, and E wave deceleration time were measured. Mitral valve annulus velocities in septal and lateral segments (e’ wave) were obtained by means of tissue Doppler, in apical 4-chamber view.

Apical 2-, 3- and 4-chamber windows were used to obtain 2D LV longitudinal systolic strain. It was possible to generate parametric imaging of myocardial strain by integrating an automated method. The longitudinal strain of each segment was measured and expressed using a bull's eye map. The software calculated global longitudinal strain as the average of regional strains throughout the entire LV. The 3D echocardiographic data were obtained through 6 consecutive electrocardiographically monitored beats to calculate the total volume.

The automatic tracing of the endocardial and epicardial borders made it possible to obtain ejection fraction at end-systole and end-diastole, cardiac output, sphericity index, LV mass, and the following 3D parameters of myocardial deformation: global longitudinal strain, global circumferential strain, global area strain, and global radial strain. The obtained data were exported to an EchoPAC, version 112.1.3, GE Healthcare workstation for offline analysis.

### Statistical analysis

Data were grouped into frequency tables, with absolute frequencies and their respective percentages, as well as descriptive measures (median and interquartile range [25% and 75% percentiles] for quantitative data). Normality was assessed using the Shapiro-Wilk test. Fisher's test was used to compare categorical data, and the Mann-Whitney test was used to compare quantitative variables, as they did not show normal distribution. For all tests, comparisons with p value less than 5% were considered significant. SPSS version 25.0 software was used for analyses.

## Results

None of the children presented cardiac symptoms or complaints. In one patient, a grade I systolic murmur was detected in aortic focus, with normal heart sounds. The baseline electrocardiogram demonstrated sinus rhythm in all patients. None of them presented pre-excitation syndrome. One of the children in the case group had a short Pr interval, and another had second degree right bundle branch block on the baseline electrocardiogram.

Table 1 displays the demographic and clinical characteristics of the case and control groups studied. The groups were homogeneous in terms of sex, age, weight, height, body surface, and heart rate measured during the 3D examination.

**Table 1:** Demographic and clinical characteristics according to study group

Variables		Group		p value
		Cases (n = 7)	Controls (n = 7)	
Sex				
	Female	4 (57.1%)	4 (57.1%)	>0.999^f^
	Male	3 (42.9%)	3 (42.9%)	
**Age**				
	Median (IQR)	6.0 (2.0 - 8.0)	6.0 (2.0 - 8.0)	>0.999^mw^
**Weight**				
	Median (IQR)	22.0 (13.0 - 39.0)	18.0 (13.3 - 27.0)	0.710^mw^
**Height**				
	Median (IQR)	1.20 (1.00 - 1.42)	1.10 (1.05 - 1.25)	0.805^mw^
**Body surface area**				
	Median (IQR)	0.86 (0.60 - 1.24)	0.74 (0.70 - 0.97)	0.805^mw^
**HR 3D**				
	Mediana (IQR)	56 (52 - 88)	80 (68 - 98)	0.165^mw^

f: Fisher's exact test; HR 3D: heart rate during three-dimensional echocardiogram; IQR: interquartile range; mw: Mann-Whitney test.

In the assessment of conventional echocardiographic parameters, a significant difference was observed in the anteroposterior diameter of the left atrium, diastolic thickness of the interventricular septum and the LV posterior wall, LV myocardial mass indexed by body surface area, end-diastolic volume ratio divided by total mass (EDV/M), and relative LV wall thickness (RWT) (Figure 1). For all these measurements, the median was higher in cases, except for EDV/M, which showed a higher median in the control group (Table 2).

**Figure 1:** In A) parasternal longitudinal axis of the left ventricle. In B) M-mode guided by 2-dimensional imaging, where measurements of the cardiac chambers, myocardial mass, and left ventricular systolic function were taken. AO: aorta; LA: left atrium; LV: left ventricle; IVS: interventricular septum in diastole; PW: posterior wall in diastole; RV: right ventricle; LV: left ventricle.

**Table 2:** Assessment of conventional echocardiographic parameters according to study group

Variables		Group		p value
		Cases (n = 7)	Controls (n = 7)	
**Ao**				
	Median (IQR)	23 (17 - 23)	18 (17 - 22)	0.259
**LA**				
	Median (IQR)	27 (19 - 30)	18 (16 - 23)	0.026
**RV**				
	Median (IQR)	14 (12 - 16)	12 (10 - 16)	0.383
**LVSD**				
	Median (IQR)	23 (19 - 24)	23 (20 - 26)	0.383
**LVDD**				
	Median (IQR)	38 (31 - 42)	36 (30 - 42)	0.535
**IVS**				
	Median (IQR)	8 (6 - 10)	6 (4 - 6)	0.017
**LVPW**				
	Median (IQR)	8 (6 - 9)	5 (4 - 5)	0.007
**Index**				
	Median (IQR)	1 (1 - 1.2)	1 (1 - 1.2)	0.902
**EDV**				
	Median (IQR)	61 (37 - 78)	45 (36 - 61)	0.209
**ESV**				
	Median (IQR)	18 (11 - 21)	14 (11 - 21)	0.620
**SV**				
	Median (IQR)	27 (21 - 44)	30 (22 - 43)	>0.999
**EF**				
	Median (IQR)	72 (71 - 76)	67 (62 - 76)	0.165
**FS %**				
	Median (IQR)	41 (39 - 45)	38 (33 - 42)	0.138
**LV myocardial mass**				
	Median (IQR)	103 (27.6 - 133)	30 (20.6 - 64)	0.128
**LV indexed mass**				
	Median (IQR)	96 (67.3 - 107)	43 (37.8 - 54)	0.007
**EDV/M**				
	Median (IQR)	0.59 (0.57 - 0.8)	1.1 (1.04 - 1.75)	0.004
**RWT**				
	Median (IQR)	0.42 (0.32 - 0.44)	0.28 (0.24 - 0.33)	0.007
**Mitral valve E wave**				
	Median (IQR)	1.08 (0.86 - 1.33)	1 (0.89 - 1.2)	0.620
**Mitral valve A wave**				
	Median (IQR)	0.36 (0.31 - 0.49)	0.38 (0.34 - 0.42)	0.710
**E/A mitral ratio**				
	Median (IQR)	2.7 (2.2 - 3.4)	2.48 (2.3 - 2.6)	0.902
**E wave deceleration time**				
	Median (IQR)	160 (151 - 174)	174 (152 - 180)	0.383
**Septal e’ wave**				
	Median (IQR)	12 (11 - 13)	12 (10 - 14)	0.805
**E/e’ wave ratio**				
	Median (IQR)	8.1 (7.2 - 10)	8.3 (5.2 - 8.6)	0.535
**Peak TR velocity**				
	Median (IQR)	2.28 (2.08 - 2.41)	2.3 (1.94 - 2.35)	0.556
**PASP**				
	Median (IQR)	26 (23 - 29)	26 (20 - 27)	0.413

Ao: aorta; EDV: left ventricular end-diastolic volume; EF: ejection fraction; ESV: end-systolic volume; FS: left ventricular fractional shortening; IVS: interventricular septum thickness in diastole; LA: left atrium; LVDD: left ventricular diastolic diameter; LVPW: left ventricular posterior wall thickness in diastole; LVSD: left ventricular systolic diameter; PASP: pulmonary artery systolic pressure; RV: right ventricle; RWT: relative wall thickness; SV: stroke volume; TR: tricuspid regurgitation.

* Mann-Whitney test.

Regarding echocardiographic parameters on the 3D exam, no statistically significant difference was observed (Central Figure). The data are shown in Table 3.

**Table 3:** Assessment of 3-dimensional echocardiographic parameters according to study group

Variables		Group		p value
		Cases (n = 7)	Controls (n = 7)	
**Final diastolic volume, 3D**				
	Median (IQR)	45 (24 - 57)	42 (26 - 59)	>0,999
**Final systolic volume, 3D**				
	Median (IQR)	17 (10 - 22)	16 (11 - 24)	>0,999
**Stroke volume, 3D**				
	Median (IQR)	27 (15 - 35)	26 (16 - 35)	>0,999
**Ejection fraction, 3D**				
	Median (IQR)	62 (60 - 63)	62 (59 - 63)	>0,999
**Cardiac output, 3D**				
	Median (IQR)	1,5 (1,3 - 2)	1,6 (1,5 - 2,1)	0,456
**Myocardial mass, g, 3D**				
	Median (IQR)	80 (30 - 96)	54 (31 - 60)	0,165
**Myocardial mass, g/m2, 3D**				
	Median (IQR)	87,7 (59 - 112)	61 (44 - 68)	0,165
**Sphericity index, 3D**				
	Median (IQR)	0,51 (0,45 - 0,59)	0,6 (0,56 - 0,6)	0,165

3D: three-dimensional echocardiogram; IQR: interquartile range.

In relation to the myocardial deformation indices obtained by speckle tracking, no statistically significant changes were detected on the 2D or 3D modality (Table 4, Figure 2).

**Table 4:** Myocardial deformation indices on 2- and 3-dimensional speckle tracking

Variables		Group		p value
		Cases (n = 7)	Controls (n = 7)	
**GLS 3D**				
	Median (IQR)	18 (17 - 21)	19 (18 - 21)	0.259
**GRS 3D**				
	Median (IQR)	51 (47 - 56)	52 (48 - 56)	0.620
**GCS 3D**				
	Median (IQR)	17 (15 - 28)	20 (18.8 - 21)	0.128
**Area strain 3D**				
	Median (IQR)	31 (29 - 34)	32 (30 - 36)	0.259
**3-chamber view, 2D**				
	Median (IQR)	18.3 (18.1 - 20.4)	19.2 (19.1 - 22.4)	0.097
**4-chamber view, 2D**				
	Median (IQR)	19.9 (19.1 - 20.1)	20.1 (19.6 - 20.3)	0.318
**2-chamber view, 2D**				
	Median (IQR)	21.4 (20.8 - 24.9)	22.8 (21.2 - 23.8)	0.710
**GLS 2D**				
	Median (IQR)	19.7 (19.3 - 21)	20.7 (20.2 - 22.6)	0.318

GCS 3D: global circumferential strain on three-dimensional echocardiogram; GLS 2D: global longitudinal strain on two-dimensional echocardiogram; GLS 3D: global longitudinal strain on three-dimensional echocardiogram; GRS 3D: global radial strain on three-dimensional echocardiography; IQR: interquartile range.

**Figure 2:** In A) bull's eye parametric map of longitudinal systolic strain obtained on the 2-dimensional exam. In B) 3-dimensional exam, where end-diastolic and end-systolic volumes, ejection fraction, cardiac output, and sphericity index were obtained.

## Discussion

In 2001, Gollob et al.,^1^ described the *PRKAG2* gene as the cause of the syndromic association between LV hypertrophy, pre-excitation, progressive deterioration of the conduction system, and sudden death; since then, accumulated reports from the past two decades have made it possible to recognize the wide scope of clinical manifestations that can be associated with *PRKAG2* syndrome, as diverse as myalgias, seizures, syncope, progressive atrioventricular block, and heart failure.^6,7,11^

Nonetheless, the progression to frank heart failure during the first years of life represents an exception.^7^ In the majority of cases described, asymptomatic evolution or discrete and non-specific symptoms were observed until the end of adolescence and the beginning of adulthood.^6^

*PRKAG2* syndrome results from an autosomal dominant mutation, which is familial in nature. It leads to AMPK dysregulation, with consequent metabolic dysregulation that leads to progressive glycogen accumulation. Its occurrence is rare, and correct diagnosis requires genetic study and extensive cardiological assessment.^11,12^

The electrocardiographic appearance of this disease shows short Pr interval in most cases, right bundle branch block, and atrioventricular or sinoatrial blocks. High voltage QRS complexes with ventricular repolarization abnormalities can be observed, even in the absence of LV hypertrophy on echocardiography.^13^

The characterization of echocardiographic changes expressed over the course of the disease in adults has been researched.^12,13^ In addition to the use of conventional echocardiography, the aid of 3D modality and the assessment of myocardial deformation allowed better interpretation of the progressive changes in cardiac morphology and function that are associated with the course of the disease.^8,14,15^ However, the focus of the majority of studies that used echocardiography has been on adult patients, with already evident manifestations of the disease.

Taking into account the autosomal dominant nature of the *PRKAG2* mutation, the cumulative and progressive nature resulting from AMPK dysfunction, and the natural course of the disease, it is reasonable to assume that echocardiographic changes resulting from glycogen accumulation begin in childhood, generating indicators that are increasingly evident of the progressive course of this entity.

The present study aimed to compare echocardiographic studies of children whose patients were diagnosed with *PRKAG2*, between the ages of 9 months and 12 years, paired with healthy individuals from the control group. Care was taken to pair research participants with individuals of the same sex. Considering the relative scarcity of standardized references in echocardiographic measurements of children, especially when considering measurements obtained using advanced techniques, this pairing aimed to minimize differences that did not result from cardiac manifestations of *PRKAG2* mutation. The small sample size was due to the rarity of the disease.

On conventional echocardiography, measurements of the left atrium, interventricular septum, LV posterior wall, indexed mass, and RWT were greater than those found in the control group, demonstrating statistical significance (p < 0.05).

The findings described above corroborate the expected trend that, in this disease characterized by the progressive accumulation of glycogen in cardiomyocytes, changes related to the progression to a pattern of ventricular hypertrophy can already be found many years before the clinical manifestation of the disease.^16^ Furthermore, the role of echocardiography, a non-invasive and widely available tool, in the follow-up of these patients may prove viable, with the aim of anticipating clinical manifestations in children of patients affected by *PRKAG2*.

Although 3D echocardiography and the assessment of cardiac deformation indices have already been demonstrated as relevant complementary tools to conventional echocardiography analysis, in our study, we did not detect significant changes in relation to the control group. A possible explanation would be that 2D-guided M-mode has greater temporal resolution, in addition to being an effective modality in recording multiple cardiac cycles.^17^

The small sample size, the cross-sectional nature of the study, the rarity of the disease, and the heterogeneity of phenotypes related to *PRKAG2* syndrome, in addition to the fact that there are few studies of reference values for children using new echocardiographic technology, constitute study limitations.

## Conclusion

Children with pathogenic variants in *PRKAG2* and children of parents with the same disease already showed a tendency toward increased LV wall thickness in relation to the control group. Echocardiographic follow-up, beginning in childhood, of children whose parents are affected by *PRKAG2* syndrome can be useful in anticipating disease manifestations, allowing better planning of therapeutic follow-up and prognosis. Larger longitudinal studies focusing on children are needed to better understand the disease evolution and identify useful echocardiographic findings for follow-up of this special group of patients.
